# Magnetic resonance imaging of in vitro urine flow in single and tandem stented ureters subject to extrinsic ureteral obstruction

**DOI:** 10.1111/iju.14942

**Published:** 2022-06-01

**Authors:** Ishai Dror, Talia Harris, Vyacheslav Kalchenko, Yaniv Shilo, Brian Berkowitz

**Affiliations:** ^1^ Department of Earth and Planetary Sciences Weizmann Institute of Science Rehovot Israel; ^2^ Department of Chemical Research Support Weizmann Institute of Science Rehovot Israel; ^3^ Department of Veterinary Resources Weizmann Institute of Science Rehovot Israel; ^4^ Department of Urology, Kaplan Medical Center Affiliated with the Hebrew University Rehovot Israel

**Keywords:** drainage, flow dynamics, noninvasive measurement, phantom kidney

## Abstract

**Objective:**

To quantify the relative volumetric flows in stent and ureter lumina, as a function of stent size and configuration, in both unobstructed and externally obstructed stented ureters.

**Methods:**

Magnetic resonance imaging was used to measure flow in stented ureters using a phantom kidney model. Volumetric flow in the stent and ureter lumina were determined along the stented ureters, for each of four single stent sizes (4.8F, 6F, 7F, and 8F), and for tandem (6F and 7F) configurations. Measurements were made in the presence of a fully encircling extrinsic ureteral obstruction as well as in benchmark cases with no extrinsic ureteral obstruction.

**Results:**

Under no obstruction, the relative contribution of urine flow in single stents is 1–10%, while the relative contributions to flow are ~6 and ~28% for tandem 6F and 7F, respectively. In the presence of an extrinsic ureteral obstruction and single stents, all urine passes within the stent lumen near the extrinsic ureteral obstruction. For tandem 6F and 7F stents under extrinsic ureteral obstruction, relative volumetric flows in the two stent lumina are ~73% and ~81%, respectively, with the remainder passing through the ureter lumen.

**Conclusions:**

Magnetic resonance imaging demonstrates that with no extrinsic ureteral obstruction, minimal urine flow occurs within a stent. Stent lumen flow is significant in the presence of extrinsic ureteral obstruction, in the vicinity of the extrinsic ureteral obstruction. For tandem stents subjected to extrinsic ureteral obstruction, urine flow also occurs in the ureter lumen between the stents, which can reduce the likelihood of kidney failure even in the case of both stent lumina being occluded.

**Abbreviations & Acronyms**
CT: computed tomography
EUO: extrinsic ureteral obstruction
MRI: magnetic resonance imaging

## Introduction

Drainage of obstructed kidneys, whether caused by occluding ureteral stones or either intrinsic ureteral obstruction or EUO, is required to preserve renal function. Ureteral stents are employed frequently to facilitate drainage in the urinary tract. However, the choice of stent size and configuration (single, tandem) remains largely subjective, with little quantitative information on the drainage characteristics of stent size and configuration, and conflicting recommendations and practices regarding use of single and tandem stents with a range of diameters.

Few studies have focused on quantitative analysis of the impact of stent size and configuration (single, tandem) on volumetric flow in ureteral stents, whether in vivo,^1^ ex vivo,^2^ in vitro,^3^, ^4^ or in silico,^5^, ^6^, ^7^, ^8^ in the context of EUO or occlusion by ureteral stones. Moreover, tandem stent configurations have received particular attention as a potentially significant means to ensure drainage in the presence of EUO,^9^, ^10^, ^11^ yet discussion continues over the preferred use of single or tandem configurations.

The goal of this work was to quantify the (cross‐sectional) velocity distribution and thus relative volumetric flow rates in the stent and ureter lumina of stented ureters, both without and subject to an EUO. The in vitro experiments incorporated a realistic phantom kidney model to represent the renal pelvis and considered the impact of single and tandem stent configurations with stents of different diameters. Volumetric flow rates at three locations along each stented ureter were determined by high‐resolution measurements using MRI.

## Methods

### Phantom kidney model

**FIGURE 1:**
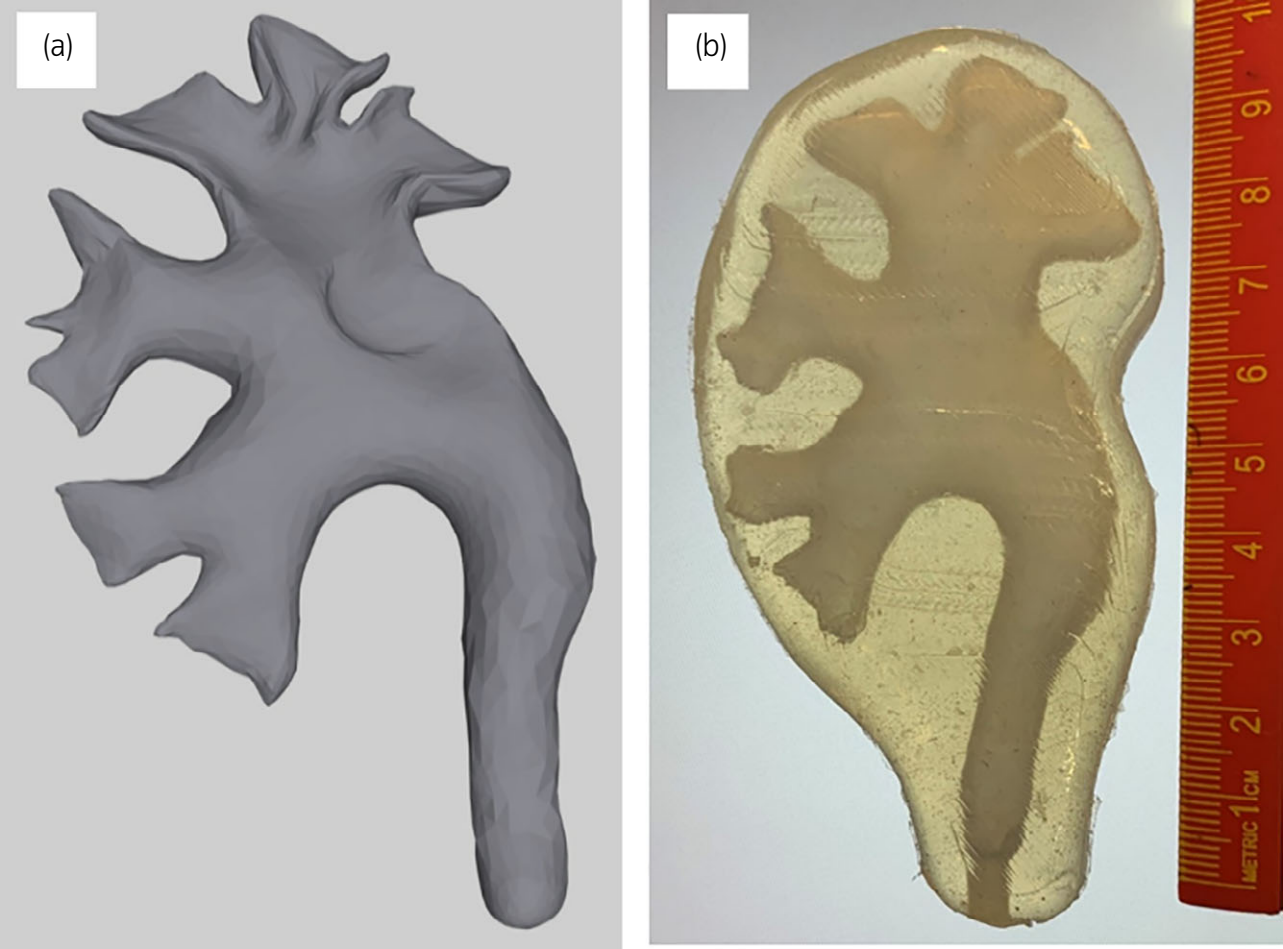
(a) 3D rendition of renal pelvis and calyxes based on human CT, and (b) phantom kidney model; ~5 cm wide, ~9.5 cm high, ~1.5 cm thick. [Colour figure can be viewed at wileyonlinelibrary.com]

A phantom kidney model was developed for the in vitro laboratory experiments. The model represents the renal collecting system, which is the region of interest in terms of fluid volume and stent placement. The model was based on CT images and data from a human abdomen and urinary tract, freely available from two standard libraries.^12^, ^13^ This information was used to develop a 3D reconstruction of the renal calyxes and pelvis, which was then printed in 3D and used to produce a wax model. The wax model was placed in a kidney shaped mold, and silicon was injected. After curing of the silicon, the wax was liquefied out of the kidney unit. Figure 1 shows the detail of the calyxes and the renal pelvis and the resulting phantom kidney model.

### Kidney‐ureter‐stent‐bladder system

**FIGURE 2:**
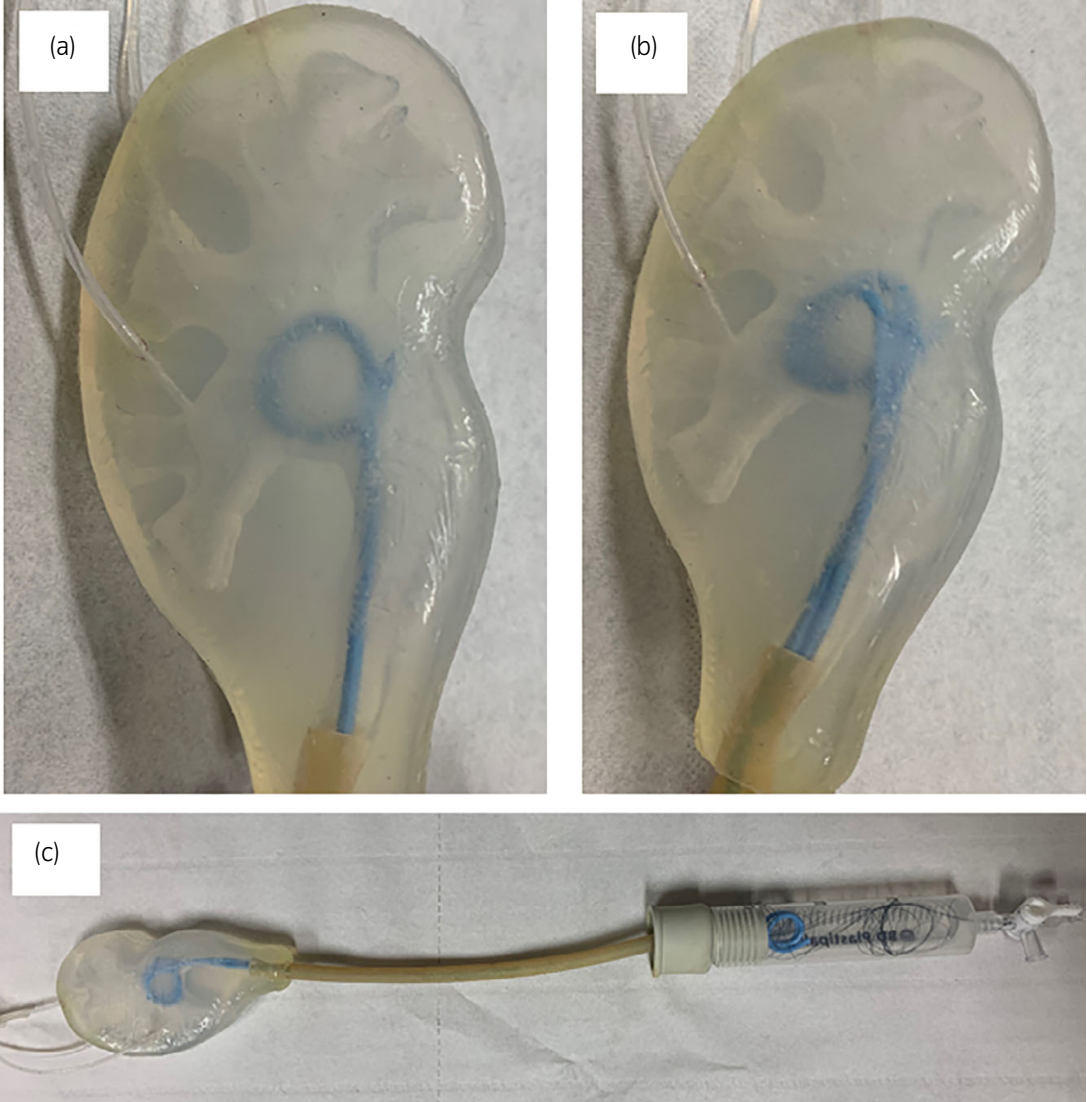
Experimental setup showing the phantom renal pelvis (a) with single 6F stent, (b) with tandem 6F stent, and (c) with the full stented ureter and bladder system. Note the three injection tubes into the (three) major calyxes are visible. [Colour figure can be viewed at wileyonlinelibrary.com]

To simulate urine flow dynamics in a stented ureter, a kidney‐ureter‐bladder system was constructed using the phantom kidney model, connected to latex tubing representing a flexible ureter^3^, ^4^ (NEWTEX; NewAge Industries Inc, Southampton, PA, USA). Tubing with 4.76 mm inner diameter (wall thickness 0.79 mm) was chosen to facilitate emplacement of tandem stents. Six stented ureter systems were considered – 4.8F, 6F, 7F, 8F, tandem 6F, tandem 7F – using double‐J (pigtail) stents (Percuflex Plus; Boston Scientific, Marlborough, MA, USA). The tubing was connected to a plastic vessel representing a bladder. The kidney model and bladder vessel accommodated full expansion of the proximal and distal stent pigtails. An artificial urine solution^14^ was employed; flow rates were controlled by a syringe pump (KD Scientific Inc., Holliston, MA, USA) with flow divided equally into three inlet tubes inserted into the three major calyxes. Figure 2 shows the experimental setup, including single 6F and tandem 6F stent pigtails in the phantom kidney, together with the full stented ureter and bladder system.

Urine flow in the kidney‐ureter‐stent‐bladder system, for each stent configuration, was investigated for two cases – with no EUO and with a fully encircling EUO (complete ureter lumen occlusion) – located in the middle of the ureter. The precise structure of EUOs is generally uncertain, so a worst‐case situation leading to complete obstruction of the ureter lumen was chosen here. The EUO was simulated by attaching three nylon, self‐locking cable‐zip ties, each of 3 mm width, to form a ~1.3 cm long region that constricted ureter lumen.

### MRI measurements

MRI measurements of fluid flow were performed on each of the six stented ureter configurations (4.8F, 6F, 7F, 8F, tandem 6F, and tandem 7F), at each of two overall volumetric flow rates, namely 30 and 50 mL/h. Steady‐state fluid velocities in cross‐sections perpendicular to the stented ureters were measured at three locations – mid‐ureter, and ~4 cm proximal and distal to the mid‐ureter – both without and with an EUO placed at the mid‐ureter measurement location. At each location, three measurements were made over a length of ~1.2 cm and averaged. Efforts were made to select measurement locations removed from the stent side holes. The variability (standard deviation) among these measurements was generally <±1%; each cross‐sectional slice, 1 mm thickness, had an in‐plane resolution of 50 × 50 μm. Fluid velocity measurements were then converted to volumetric flow rates in each of the stent (single or tandem) and ureter lumina; the sum of these flow rates was confirmed to match the imposed total flow in the system (30 or 50 mL/h).

Details on the MRI measurement and analysis protocols are given in Appendix S1.

## Results

**FIGURE 3:**
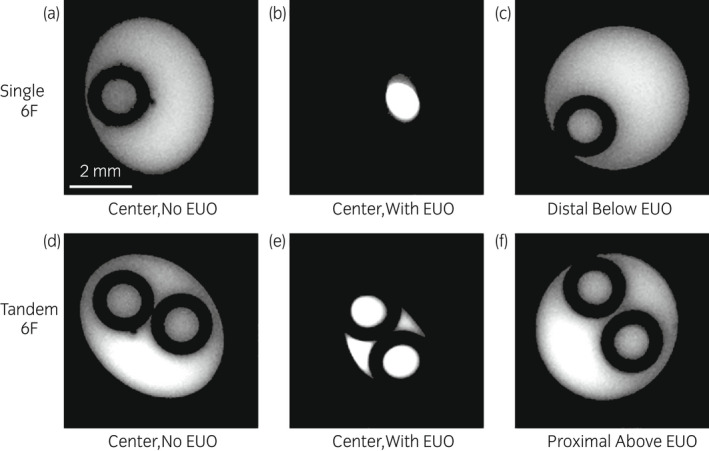
Representative MRI stented ureter cross‐sections showing the stent(s) within the ureter lumen, without and with EUO. Colors: Black represents no flow regions (stent walls and border of ureter walls); white/gray represents fluid velocity, normalized within each image, with higher intensity of white representing higher velocity. (a) Single 6F, no EUO; (b) single 6F, with EUO; (c) single 6F, distal to the EUO; (d) tandem 6F, no EUO; (e) tandem 6F, with EUO; and (f) tandem 6F, proximal to the EUO. [Colour figure can be viewed at wileyonlinelibrary.com]

**FIGURE 4:**
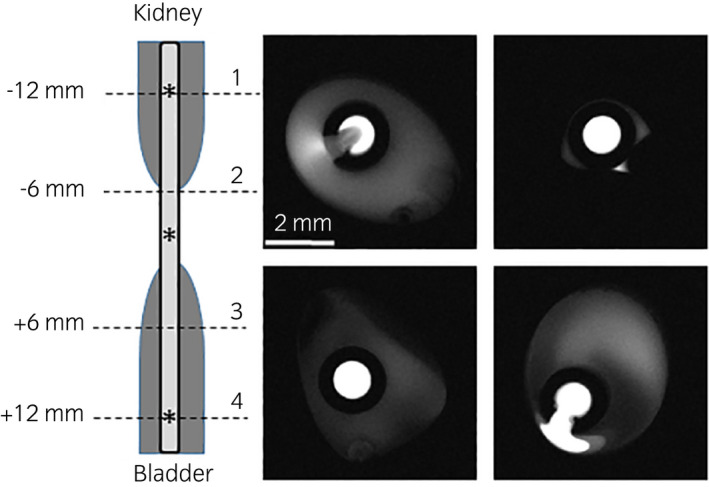
Representative MRI cross‐sections showing flow through side holes proximal (1) and distal (4) to the EUO (2, 3). Images are for a single 6F stent. Images (1) and (4) show urine velocities in stent and ureter lumina, and as well within the connecting stent side hole; images (2) and (3) show relative urine velocities in the stent lumen, which in this region conduct 100% of the flow. *Indicates location of a stent side hole. [Colour figure can be viewed at wileyonlinelibrary.com]

Representative MRI cross‐sections of the fluid velocity in the mid‐ureter regions of the stented ureter, without and with EUO, are shown in Figure 3. Comparing Figures 3a,b, it is seen that, as expected, essentially all of the fluid is forced through the stent lumen in the region of the EUO. In contrast, a tandem stent configuration in the EUO region enables flow in small region(s) in the ureter lumen between the tandem stents (Fig. 3d,e). In all cases, the major role of the stent(s) is to facilitate fluid flow (drainage) in the vicinity of the EUO. Distal (Fig. 3c) and proximal (Fig. 3f) to the EUO, the majority of the flow occurs in the ureter lumen, with the side holes enabling fluid communication between ureter and stent lumina. The role of side holes is seen clearly in Figure 4, which enables urine in the ureter lumen to bypass the EUO, through the stent, and then reenter the ureter lumen further downstream.

The volumetric flow rates for all of the kidney‐ureter‐stent‐bladder systems, determined from the MRI measurements, are summarized in Table 1. The results are presented as the % measured volumetric flow rate in the stent lumen relative to the total (combined ureter‐stent lumina) volumetric flow rate. Also shown in Table 1, for comparison, is the theoretical ratio (%) between the cross‐sectional stent lumen area and the sum of stent and ureter cross‐sectional lumina, in the absence of any EUO. Results shown in Table 1 are for a total flow rate of 30 mL/h; the results for a total flow rate of 50 mL/h were essentially the same, with ±1% maximum variability in all cases. For comparison, theoretical, relative ratios of the stent lumen area, for perfectly circular ureter and stent(s), are also shown. The finding that volumetric flow rate has no notable effect on the results shown in Table 1 is expected because the relative stent/ureter lumina cross‐sectional areas are constant, and volumetric flows increase essentially linearly with increases in flow rate or overall pressure gradient. Moreover, there is essentially no variation in relative volumetric flow rates in the stent and ureter lumina along the length of a stented ureter (proximal, mid‐ureter, distal) when no EUO is present.

**TABLE 1:** Comparison of % measured flow rates in different stent and EUO configurations

Stent size and configuration	Theoretical ratio (%) of cross‐sectional stent lumen area to sum of stent lumen area and functional ureter lumina, in the absence of EUO†	% volumetric flow in stent(s), for total ureter‐stent flow of 30 mL/h			
		No EUO	Mid‐ureter EUO		
		Mid‐ureter (%)‡	Proximal (%)§	Mid‐ureter (%)	Distal (%)
Single 4.8F	6	1	6	100	5
Single 6F	7	1	4	100	8
Single 7F	12	5	7	100	5
Single 8F	18	10	26	100	15
Tandem 6F	16¶	6	8	73††	7
Tandem 7F	28¶	28	33	81††	36

† Functional ureter lumen cross‐sectional area = internal ureter lumen area minus the external cross‐sectional areas (lumen and walls) of the stent(s). Note that stent and ureter cross‐sections are assumed to be exactly circular.

‡ Essentially identical values (±2%) were determined also for proximal and distal locations ~4 cm from the mid‐ureter location.

§ Proximal and distal measurements are centered at a distance of ~4 cm on either side of the EUO positioned at the mid‐ureter location.

¶ Total for the two stents.

†† Tandem stent configuration in the EUO region enables flow in small region(s) in the ureter lumen between the tandem stents (Fig. 3d,e).

It is seen clearly that for the case of no EUO, most of the urine passes through the ureteral lumen, while only minimal flow occurs through the stents. In fact, the increase in stent diameter adds only minimal contribution of flow volume from 1% for 4.8F and 6F to 10% for 8F (Table 1). This is not surprising given that the relative ratio of stent lumen to ureter lumen changes only minimally with an increase in stent diameter. For tandem stents, the use of two 7F stents increases the stent lumen to ureter lumen ratio to nearly 30%, which translates to nearly 30% of urine flow through the combined stent lumina.

For the cases with EUO, the relative volumetric flow rates in single stents range from ~5% for the 4.8F stent to ~15–26% for the 8F stent (Table 1), in proximal and distal regions of the ureter, while ~100% of the fluid passes through the stent lumen in the vicinity of the EUO. Comparing proximal and distal locations, for different stent sizes and configurations, it is seen (Table 1) that the relative flow rates proximal and distal to the EUO are similar. In the case of tandem stents, relative volumetric flows in the two stent lumina are ~7–8% and ~33–36%, respectively, for the tandem 6F and 7F stents, in both proximal and distal regions of the ureter, while ~73–81% of the fluid passes through the stent lumina in the vicinity of the EUO, with the remainder passing through the ureter lumen that remains patent. As for the no EUO cases, the overall flow rate from the kidney (30 or 50 mL/h) has no notable effect on the relative volumetric flow rates.

Flow behavior in tandem stent configurations with EUO is significantly different than for the single stent cases. From Table 1, the relative volumetric flows in the 6F and 7F tandem stent lumina are only 73% and 81%, respectively. Recalling Figure 3, the remainder of the urine (27%, 19%, respectively) advances through the ureter lumen, which remains open notwithstanding the fully encircling EUO. To consider further, the calculated, cross‐sectional areas of tandem stent lumina directly from MRI images, such as shown in Figure 3e, are ~66% and ~72% for the tandem 6F and 7F cases, respectively, with the remaining percentages representing the cross‐sectional areas of the ureter lumina between the stents.

## Discussion

To the best of our knowledge, this is the first study to use high‐resolution MRI to quantify volumetric flow of urine in a kidney‐ureter‐bladder model, accounting for different stent sizes with and without external ureteral obstruction.

Overall, even in the presence of an EUO, stents are relatively poor urine conductors in proximal and distal regions. In such cases, volumetric flow rates vary among the proximal, mid‐ureter, and distal locations, with the stent(s) carrying most or all of the urine in the vicinity of the EUO, and with similar flow rates proximal and distal to the EUO (Table 1). Moreover, in essentially all cases, the volumetric flow rates are significantly different than the theoretically determined ratios of cross‐sectional stent lumen area to the sum of stent and ureter lumina; on the other hand, the proximal and distal relative volumetric flow rates are similar to those for the no EUO case. There is of course variation in relative flow rates along the length of the stented ureter, due to changes in stent curvature and stent contact with ureter walls (which depend also on stent size and configuration), and because the precise locations of side holes – and their proximity to the EUO – vary in each case. As seen in Figure 4, and reported in the literature,^5^ the side holes play a major role in pressure equilibration and transfer of urine between ureter and stent lumina.

In the clinical setting, the question of defining the ideal drainage option for patients with EUO has always been a subject of debate. While some urologists prefer single large diameter stent,^15^ others use tandem stents^9^, ^10^, ^11^ or metal stents.^16^, ^17^ The rationale for using tandem stents has relied on the theoretical advantage of two factors – one is the fact that two stents offer twice the cross‐sectional area of single stent lumina, and the second is that a lumen is established that lies between the stents and the ureter wall (as seen in Fig. 3e). The measurements presented here show, for the first time, that the ureter lumen between the tandem stents can indeed serve as another significant pathway for urine to drain in the vicinity of the EUO. While single stent patency relies completely on stent diameter, the tandem stents allow another pathway – between the stents – for urine to flow and reduce the likelihood of kidney obstruction. The measurements in Table 1 suggest that 27% and 19% of the volumetric flow of urine, for tandem 6F and 7F stents respectively, drains through the ureter lumen between the stents.

Notwithstanding these measurements, the findings reported here cannot indicate which option – stent size and configuration – will be most effective under real, clinical EUO conditions, as other components play a role in the mechanism of stent failure, such as urine colloids, stent resistance to pressure, and tendency for occurrence of stent encrustation. Indeed, the patency of different sizes of single stents and tandem stents subjected to EUO has been examined in previous in vitro studies, showing that the 8F single stent remained patent while smaller stent sizes, tandem 6F and 7F configurations, and also metal stents all failed;^4^ failure was generally due to colloid deposition^3^, ^4^ rather than stent compression. On the other hand, a computational fluid dynamics study^7^ demonstrates that failure of a stented ureter under EUO occurs only with closure of the entire ureter lumen and more than 90% of the stent lumen. In other words, even a small path in between stent lumina will be sufficient to avoid kidney failure.

The use of tandem stents as shown in this study offers a benefit of a robust ureter lumen between tandem stents that can contribute to avoiding kidney failure. This benefit should be judged cautiously for two main possible complications. It is clear from previous studies that larger stent diameters are associated with greater irritation and pain‐related symptoms.^18^ In addition, in patients with EUO, stents are generally placed for long periods of time and are therefore prone to encrustation and occlusion of the ureter and stent lumina as well as complex procedures to change the stents.

As in all in vitro experiments, the model employed here has limitations. The duration of the experiment did not allow natural processes such as encrustation and debris accumulation to take place both in the stent and ureter lumina. The scope of this study was to accurately determine the relative volumetric flow rates in ureter and stent lumina, using high‐resolution MRI, rather than to consider long‐term processes. In this context, the particular artificial urine solution used here does not mimic real‐life scenarios in which inflammatory and infection events affect urine composition, which may in turn impact the patency of the stents and ureter lumina. Finally, it is noted that the latex tubing does not account for the effects of peristalsis; lack of peristalsis can be justified given that stenting tends to result in significant reduction of ureteral peristalsis,^19^ while longer term emplacement can allow extracellular collagen to accumulate in dilated ureters and lead to increased wall stiffness and reduced distensibility.^20^ Moreover, while indwelling stents also affect ureter dilatation, the choice of a relatively wide diameter latex tube that is somewhat larger than a normal, unstented ureter can be considered to reflect dilatation.

In conclusion, with no EUO, most urine will pass in the ureter lumen while only minimal urine flow occurs within a stent. Stent lumen flow is significant only in the presence of EUO, and in this case, only in the vicinity of the EUO. For tandem stents subjected to EUO, however, urine flow also occurs in the ureter lumen between the stents, which can therefore reduce the likelihood of kidney failure even in the case of both stent lumina being occluded. Limitations of medical MRI resolution, as well as ethical considerations, prevent in vivo measurements of ureter‐stent lumina flow and testing risks of stent and/or kidney failure even in porcine models. However, the findings of the current study together with the existing literature suggest that clinical practice should focus on use of larger (7F, 8F) diameter and tandem stent configurations, to enable their future comparison in terms of patency, durability, and patient comfort. These findings can shape future studies to further improve understanding of stent flow dynamics, and, ultimately, clinical application and management of ureteral stent use.

## Author contributions

Ishai Dror: Conceptualization; data curation; formal analysis; investigation; methodology; validation; writing – review and editing. Talia Harris: Data curation; formal analysis; investigation; methodology; software; validation; visualization; writing – original draft; writing – review and editing. Vyacheslav Kalchenko: Investigation; methodology; resources; writing – original draft; writing – review and editing. Yaniv Shilo: Conceptualization; formal analysis; investigation; methodology; validation; writing – original draft; writing – review and editing. Brian Berkowitz: Conceptualization; data curation; formal analysis; funding acquisition; investigation; methodology; project administration; resources; supervision; validation; visualization; writing – original draft; writing – review and editing.

## Conflict of interest

None declared.

## Approval of the research protocol by an Institutional Reviewer Board

N/A.

## Informed consent

N/A.

## Registry and the Registration No. of the study/trial

N/A.

## Animal studies

N/A.

## Supporting information


**Appendix S1.** MRI protocols.
