# Supplementary material for: Magnetic resonance imaging of in vitro urine flow in single and tandem stented ureters subject to extrinsic ureteral obstruction
Source: Int J Urol. 2022 Jun 1;29(10):1221–6. doi: 10.1111/iju.14942 (PMC9796179; doi:10.1111/iju.14942)
Supplement: Supplementary file 1 — Appendix S1. MRI protocols. [file IJU-29-1221-s001.docx]

**SUPPLEMENTAL MATERIALS**

**Magnetic resonance imaging of *in vitro* urine flow in single and tandem stented ureters subject to extrinsic ureteral obstruction**

Ishai Dror^1^, Talia Harris^2^, Vyacheslav Kalchenko, Yaniv Shilo^4^, Brian Berkowitz^5^

**1. Department of Earth and Planetary Sciences, Weizmann Institute of Science, Rehovot 7610001, Israel (**[ishai.dror@weizmann.ac.il](mailto:ishai.dror@weizmann.ac.il)  **Phone: +972-8-9342098 Fax: +972-8-9344124)**

**2. Department of Chemical Research Support, Weizmann Institute of Science, Rehovot 7610001, Israel (**[talia.harris@weizmann.ac.il](mailto:talia.harris@weizmann.ac.il)  **Phone: +972-8-9349294)**

**3. Department of Veterinary Resources, Weizmann Institute of Science, Rehovot 7610001, Israel (**[a.kalchenko@weizmann.ac.il](mailto:a.kalchenko@weizmann.ac.il) **Phone: +972-8-9343228 Fax: +972-8-9344133)**

4. Department of Urology, Kaplan Medical Center, Affiliated with the Hebrew University, Rehovot 7661041 **Israel (**[drshiloy@gmail.com](mailto:drshiloy@gmail.com) **Phone: +972-8-9441642 Fax: +972-8-9441344)**

**5. Department of Earth and Planetary Sciences, Weizmann Institute of Science, Rehovot 7610001, Israel (**[brian.berkowitz@weizmann.ac.il](mailto:brian.berkowitz@weizmann.ac.il) **Phone: +972-8-9342098 Fax: +972-8-9344124)**

***MRI measurement protocol and data processing***

MRI flow measurements were performed on a horizontal Biospec 15.2 T USR preclinical MRI scanner with an Avance IIIHD console equipped with a 35 mm 1H quadrature volume coil operating with Paravision 6 (Bruker Biospin, Ettlingen, Germany). 2D Gradient Recalled Echo (GRE) images with 50 μm × 50 μm in-plane resolution (1 mm slice thickness) were acquired perpendicular to the ureter axis with the following acquisition parameters: FA=90^o^, TR=100 ms, TE=3 ms, BW=50 kHz, matrix size 160 × 160, 10 averages; including 12 s of dummy scans to achieve steady state, the acquisition time was ≈3min. Before acquisition of each set of images, the sample was automatically shimmed and the 90^o^ pulse was determined,

For each position, three slices were imaged: first in an interleaved fashion in the absence of flow and then individually in the presence of total flow through the kidney phantom of 30 mL/h and 50 mL/h. For the acquisition parameters chosen and the range of flow rates investigated, the increase in signal is proportional to the flow velocity in the direction perpendicular to the imaging plane, as confirmed by measuring flow through a tube for the range of flow rates observed.

Flow maps were calculated with custom written Matlab scripts in the following manner: first, images were masked to remove voxels not containing liquid (i.e., background and tubing walls). For the remaining voxels, flow maps were determined by dividing the voxel intensity in the presence of flow to the average voxel intensity in the absence of flow, with the overall velocity across the entire imaging slice determined by the global flow rate set by the syringe pump. In order to determine the relative flow distribution in the stent, a region of interest (ROI) was manually drawn around the stent(s), and by comparing the number of voxels and the sum of velocity of the voxels in this ROI to the total number of voxels and total sum of velocity in all voxels, the volume and flow percentages, respectively, in the stent(s) was determined.
